# Adult intestinal non-rotation with chronic obstructive symptoms: a rare case report and review of diagnostic laparoscopy and functional reconstruction beyond standard Ladd’s procedure

**DOI:** 10.1093/jscr/rjaf957

**Published:** 2025-12-05

**Authors:** Dani Alokla, Sliman Marina, Mahmoud Alhamadeh Alswij, Mohammed Abdulkafee Alshareeda, Mohammad Naser Jasem Alahmad, Ali Bareaj Alkalet, Adnan Alhaj Hasan

**Affiliations:** Emergency Medicine, Damascus Hospital, Ministry of Health, Damascus, Syria; Faculty of Medicine, Damascus University, Damascus, Syria; Department of Internal Medicine, Hematology Department, Al-Mouwasat University Hospital, Damascus, Syria; General Surgery, Faculty of Medicine, Tishreen University, Latakia, Syria; Department of Gastroenterology, Faculty of Medicine, Aleppo University, Aleppo, Syria; General Surgery, Lattakia University Hospital, Faculty of Medicine, Lattakia University, Latakia, Syria; Internal Medicine, Faculty of Medicine, Damascus University, Damascus, Syria

**Keywords:** adult intestinal malrotation, non-rotation, chronic bowel obstruction, diagnostic laparoscopy, Ladd procedure, ileosigmoid anastomosis

## Abstract

Intestinal malrotation is rarely diagnosed in adulthood and often presents with non-specific or chronic symptoms. We report a case of a 54-year-old woman presenting with recurrent abdominal pain and obstructive symptoms. Preoperative imaging was inconclusive, and diagnostic laparoscopy revealed non-rotation with abnormal positioning of the bowel and a mobile transverse colon looping around the superior mesenteric artery without volvulus. A standard Ladd procedure was performed, along with a tailored ileosigmoid anastomosis to restore bowel continuity and prevent future volvulus. The postoperative course was uneventful, and the patient remained symptom-free at follow-up. Adult intestinal malrotation may require individualized surgical correction when anatomical variations are encountered intraoperatively. Prompt surgical adaptation can lead to favorable outcomes.

## Introduction

Congenital midgut malrotation is usually diagnosed in infancy, with adult presentations being very rare (0.2%–0.5% of cases) [1]. In adults, symptoms tend to be vague and intermittent, often leading to delayed diagnosis [2]. Imaging can be inconclusive, with many cases missed on X-rays, computed tomography (CT) scans, or endoscopy [3]. Surgical correction, typically via the Ladd procedure, is essential to prevent serious complications like volvulus or ischemia [4]. In some complex cases, additional procedures such as ileosigmoid anastomosis may be needed for symptom resolution. This report presents a rare adult case of intestinal non-rotation with chronic obstructive symptoms and normal imaging, successfully managed with a modified surgical approach.

## Case presentation

A 54-year-old woman with a history of four coronary catheterizations, Stage 2 chronic kidney disease, and prior pulmonary embolism presented with recurrent episodes of abdominal pain and complete constipation lasting 7–10 days. Each episode was characterized by absence of flatus and stool but without significant abdominal distension. Symptoms recurred intermittently over several months and partially responded to conservative treatment.

On examination during symptomatic periods, her abdomen was soft and non-distended, with no palpable masses or peritonitis signs. Laboratory tests revealed mild normocytic anemia (hemoglobin 10.4 g/dl) and slightly elevated C-reactive protein (8.4 mg/l), while renal function remained stable (Table 1).

**Table 1:** Lab values

Hematology—coagulation		Normal range
Protdrombin T (activity)	16.2 (77%)	13.5 (100%)
INR	1.2	
Biochemistry		
Na	134.6	134–150
K	3.72	3.5–5.0
Creatinine	0.88	0.6–1.3 mg/dl
SGPT (ALT)	19	Up to 41
Amylase	28	Up to 82
Serology		
CRP quantitative	8.4	<5 mg/l
Hematology		
Hemoglobin	10.4	12–16 g/dl
Hematocrit	34.5	35–47
RBC	3.36	4.0–5.2 × 10^6^/μl
WBC	8500	4500–11 000
Neutrophils	66	40–65
Lymphocytes	30	25–40
Monocytes	3	2–8
Eosinophils	1	0–4
Basophils	0	0–1
MCV	93	78–96
MCH	29.7	27–32
MCHC	32.1	31–35
RDW	13.2	11–16
Platelets	328 000	150 000-450 000
CKD stage—EPI creatinine	Stage II	
eGFR	78	>60 ml/min
Urea	22	

**Figure 1:**
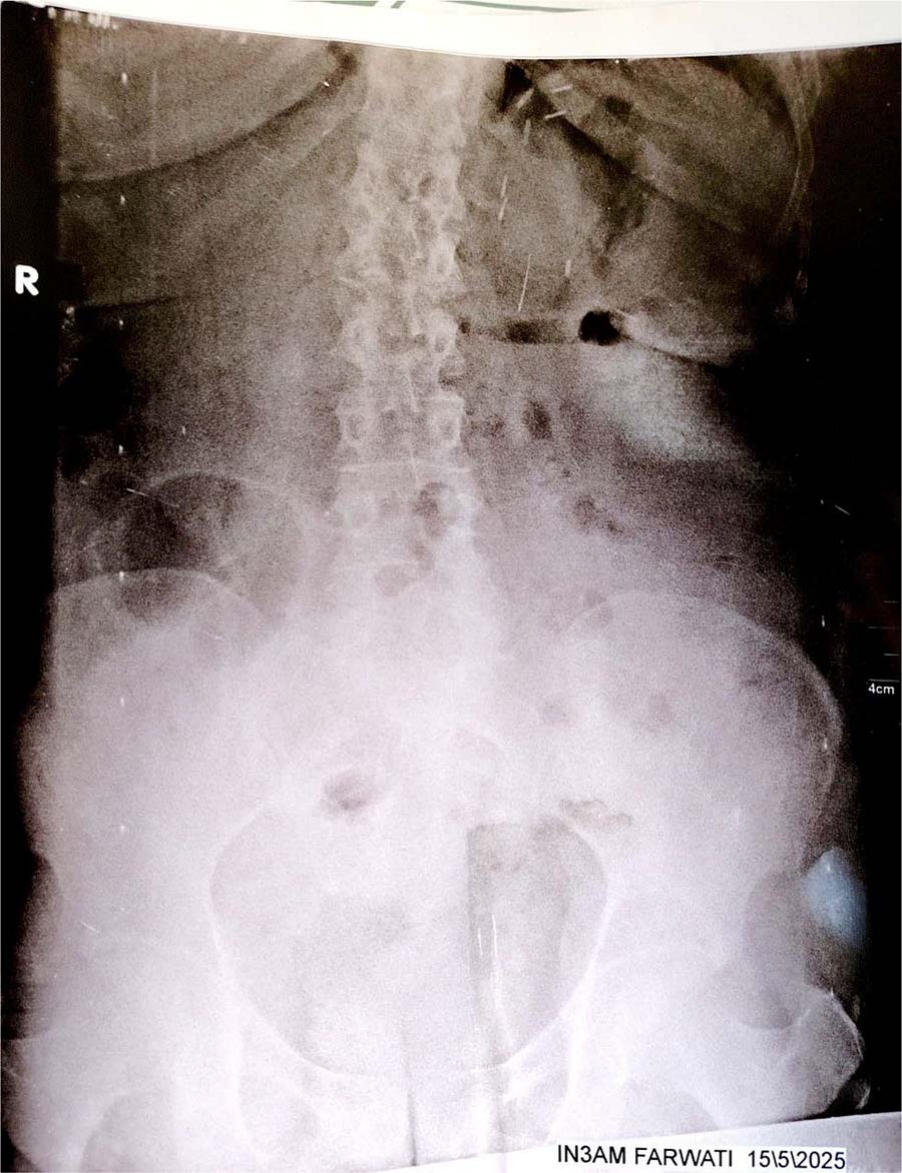
Plain abdominal X-ray (upright position) showing prominent colonic and small bowel gas patterns with no visible air-fluid levels or signs of acute obstruction. No abnormal bowel positioning or dilation is clearly evident. These findings were non-specific and contributed to the diagnostic delay.

**Figure 2:**
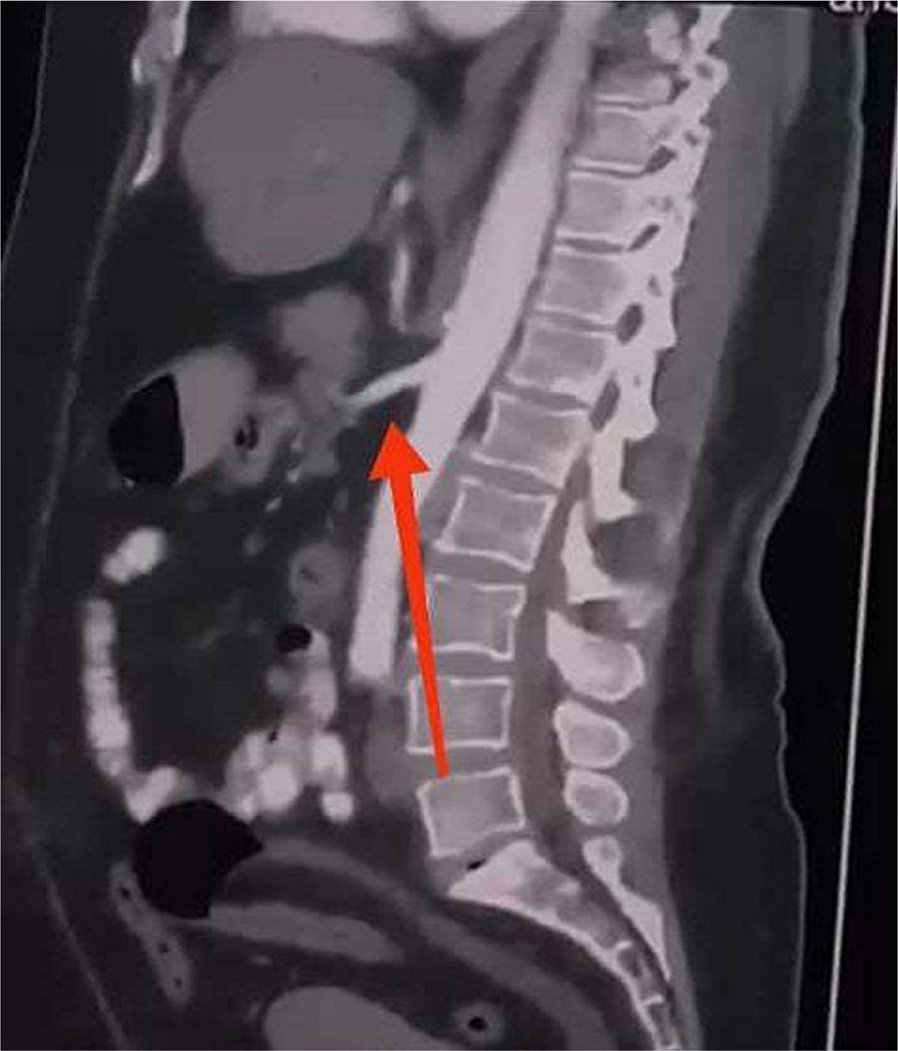
Sagittal CT scan showing a retromesenteric course of the duodenum (indicated by an arrow).

**Figure 3:**
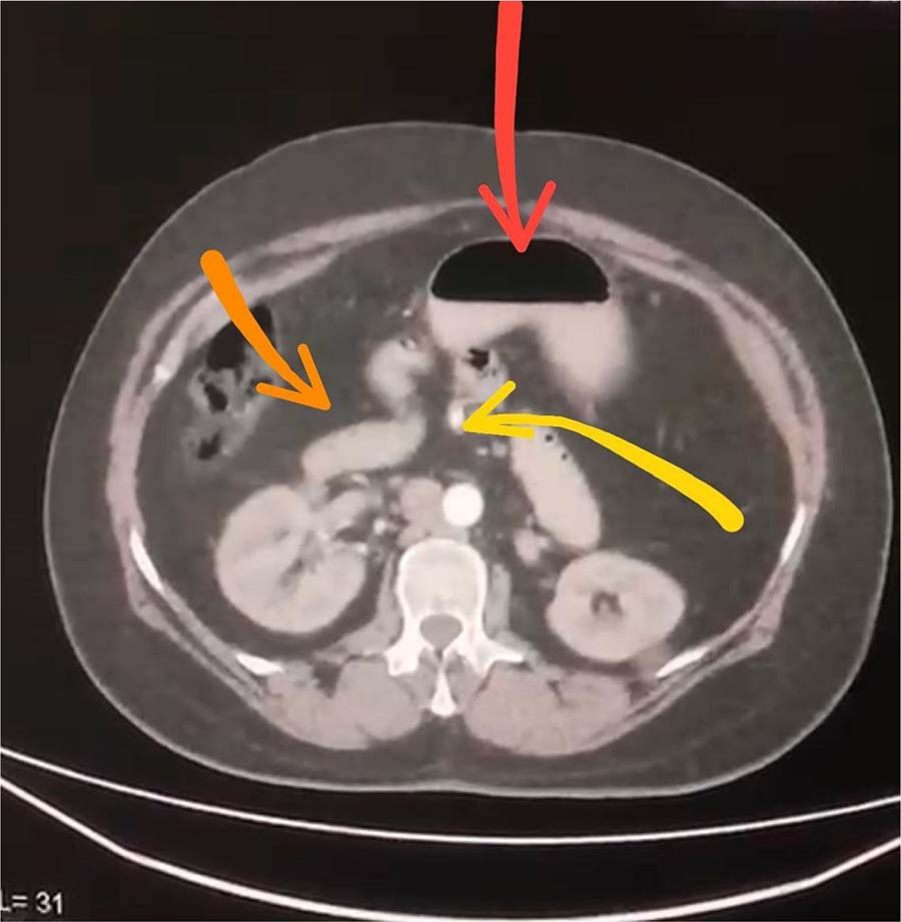
Axial contrast-enhanced CT scan demonstrating abnormal positioning of abdominal structures: The stomach is seen in the upper center (arrow), the duodenum crosses the midline anteriorly (arrow), and the superior mesenteric artery lies abnormally to the right of the superior mesenteric vein (arrow).

Initial imaging with a plain abdominal X-ray showed non-specific gas patterns without obstruction (Fig. 1). Contrast-enhanced CT revealed features of intestinal non-rotation, including abnormal duodenal positioning and atypical mesenteric vessel orientation. The sagittal view showed a retromesenteric duodenum without volvulus or bowel wall compromise (Fig. 2), while axial images demonstrated a reversed superior mesenteric artery (SMA)–superior mesenteric vein (SMV) relationship (Fig. 3), supporting malrotation. These findings are further illustrated in Supplementary Video S1), showing small bowel on the right, colon on the left, retromesenteric duodenum and abnormal SMA–SMV positioning. Colonoscopy showed normal mucosa without obstructive lesions.

Due to persistent symptoms and inconclusive imaging, diagnostic laparoscopy was performed. Intraoperatively, abnormal bowel configuration prompted conversion to an open laparotomy. The entire colon was freely mobile except for a partially fixed cecum. Notably, the transverse colon was located beneath the superior mesenteric artery and posterior to the ligament of Treitz, consistent with intestinal malrotation. There were no signs of volvulus or ischemia.

Surgical treatment involved lysis of Ladd’s bands, resection of a non-fixed segment of terminal ileum, and a primary end-to-side ileosigmoid anastomosis. The abdomen was closed primarily. An intraoperative video captured these findings and surgical steps (see Supplementary Video S2).

Postoperatively, the patient recovered well, resuming bowel function within 48 h and discharged on postoperative day four. At 1-month follow-up, she reported regular bowel movements without recurrence of symptoms. A recent clinical assessment confirmed stable condition and absence of gastrointestinal complaints.

## Discussion

Adult intestinal malrotation is a rare congenital anomaly, often presenting with vague and intermittent gastrointestinal symptoms that complicate timely diagnosis. In this case, the patient experienced recurrent episodes of complete constipation and abdominal pain over 2 years, with inconclusive imaging findings. Earlier recognition of malrotation in adults could potentially avoid prolonged morbidity by prompting diagnostic laparoscopy sooner, especially when standard imaging is nondiagnostic.

CT remains a key imaging modality, but as seen here and in prior studies, malrotation can be missed due to subtle or absent classic signs such as the “whirl” or abnormal SMA–SMV relationship [3, 5]. This underlines the importance of heightened radiologic attention to mesenteric vessel orientation and bowel positioning in adults with unexplained gastrointestinal symptoms. Colonoscopy may also fail to reveal malrotation, as it primarily evaluates the mucosa and luminal patency.

The Ladd procedure is the standard surgical treatment, involving lysis of Ladd’s bands, broadening of the mesenteric base, repositioning of bowel segments, and appendectomy [4]. However, adult cases may present with anatomical variations requiring tailored surgical approaches. In this patient, the presence of a mobile transverse colon looping beneath the superior mesenteric artery and poor bowel fixation necessitated an ileosigmoid anastomosis in addition to the standard Ladd steps to restore stable bowel continuity.

While malrotation typically presents in childhood or early adulthood, this case highlights that it can remain silent and manifest as chronic obstructive symptoms in older adults, including women in their sixth decade. Previous reports predominantly involve younger males [2, 5], making this presentation notable. Early elective intervention in such chronic cases can prevent severe complications like volvulus and ischemia seen in acute presentations [2].

Embryologically, malrotation results from arrested midgut rotation between Weeks 5 and 12 of gestation, resulting in variations like non-rotation with a short mesenteric root and absent fixation [1]. Such anatomical predispositions increase the risk of volvulus and intermittent obstruction, even in the absence of acute torsion [5]. Functional obstruction may arise due to kinking or peristaltic mismatch caused by abnormal bowel configuration, as observed in this patient.

This case underscores the diagnostic value of early laparoscopy in adults with persistent, unexplained obstructive symptoms, especially when imaging fails to clarify the cause. The favorable postoperative outcome aligns with literature reports associating early diagnosis and intervention with improved prognosis [4, 5].

See Supplementary Table S1 for a comparison with previously reported cases.

## Conclusion

Adult intestinal malrotation presenting with chronic obstructive symptoms is rare and often challenging to diagnose due to inconclusive imaging. Diagnostic laparoscopy plays a crucial role in identifying malrotation when non-invasive tests fail. Early surgical intervention with the standard Ladd procedure remains the cornerstone of treatment. However, complex anatomical variations, such as poor bowel fixation and abnormal colon positioning, may require tailored reconstructive approaches to restore stable bowel continuity and relieve symptoms. Prompt recognition and individualized surgical planning are essential to achieve optimal, durable outcomes in atypical adult presentations.

## Supplementary Material

video_2025-07-05_19-21-14_rjaf957

supp_2_rjaf957

Review_table_mal_(2)_rjaf957
