# Supplementary material for: Adult intestinal non-rotation with chronic obstructive symptoms: a rare case report and review of diagnostic laparoscopy and functional reconstruction beyond standard Ladd’s procedure
Source: J Surg Case Rep. 2025 Dec 5;2025(12):rjaf957. doi: 10.1093/jscr/rjaf957 (PMC12679281; doi:10.1093/jscr/rjaf957)
Supplement: Review_table_mal_(2)_rjaf957 [file review_table_mal_(2)_rjaf957.docx]

| **Author (Year)** | **Age/Sex** | **Presentation/Symptoms** | **Imaging Findings** | **Type of Malrotation** | **Surgical Intervention** | **Outcome** |
| --- | --- | --- | --- | --- | --- | --- |
|  |  |  |  |  |  |  |
| Mizuta et al. (2022) | 35/M | Recurrent right-sided abdominal pain (~1–2 episodes/mo) | CT: no horizontal duodenum, proximal jejunum on right side | Incomplete fixation (mesocolic hernia) | Laparoscopic Ladd’s procedure (bands divided, mesentery widened); duodenoduodenostomy for post-op duodenal stenosis | Recovered after re-op (POD 32 discharge) |
|  | 71/M | Intermittent postprandial abdominal pain (8 mo) | CT: similar malrotation pattern as above | Incomplete fixation | Laparoscopic Ladd’s procedure (as above) | Uneventful recovery (POD 10 discharge) |
|  | 58/M | Longstanding RUQ pain (10+ yr), epigastric pain, weight loss | CT: features of malrotation as above | Incomplete fixation | Laparoscopic Ladd’s procedure | Uneventful recovery (POD 7 discharge) |
| Glosser et al. (2022) | 37/F | 3-day diffuse abdominal pain; chronic recurrent pain (years) | CT: whirlpool sign of mesenteric vessels, duodenal obstruction transition | Malrotation with volvulus | Open Ladd’s procedure (division of Ladd’s bands, detorsion) | Immediate relief; asymptomatic at 2-mo f/u |
| Aregawiet al. (2025) | 18/M | Chronic crampy RUQ/epigastric pain, vomiting, constipation (months) | CT: massively dilated stomach/duodenum/jejunum; SMA/SMV whirl sign | Midgut malrotation (incomplete) | Open Ladd’s procedure (division of bands, widen mesentery, appendectomy) | Uneventful recovery; symptom-free at 3 mo |
| Aljamal et al. (2025) | 35/F | Progressive epigastric pain, recurrent vomiting | X-ray: multiple air-fluid levels; CT: whirlpool sign, SB on right/colon left | Malrotation with volvulus | Laparotomy: Ladd’s procedure; re-exploration – right hemicolectomy (cecum resection) & ileocolic stoma | Full recovery; discharged in good condition |
| Yin et al. (2024) | 45/F | Chronic vomiting (40+ years) | CT: suggestive of volvulus; laparoscopic exam confirmed malrotation | Malrotation with volvulus | Ladd’s procedure + gastrojejunostomy + Braun anastomosis | Recovered; +10 kg weight gain, symptom relief at 6-mo f/u |
| Grassi et al. (2020) | 44/F | Acute abdominal pain | CT: ileal loop conglomeration in R iliac fossa (malrotation) | Malrotation (Ladd’s band obstruction) | Laparotomy: Ladd’s (band division, adhesiolysis, Treitz fixation), small bowel resection & Meckel diverticulum removal | Unremarkable recovery |
| Islam et al. (2024) | 43/M | Nonspecific abdominal pain | CT: malrotation (duodenum right of midline) + transverse mesocolic internal hernia | Malrotation + mesocolic hernia | Laparotomy: Ladd’s procedure + repair of mesocolic hernial orifice | Uneventful recovery |
| Naddouri et al. (2024) | 18/F | Acute small bowel obstruction, bilious vomiting | CT: whirlpool sign indicating midgut volvulus | Malrotation with volvulus | Laparotomy: Ladd’s procedure (band division, adhesiolysis, appendectomy) | Good recovery (symptom resolution) |
| Akomea-Agyin et al. (2024) | 24/F | Acute on chronic abdominal pain, bilious vomiting | (CT not specified) | Malrotation + duodenal stenosis | Ladd’s procedure; relaparotomy – Heineke-Mikulicz duodenal strictureplasty | Symptom resolution after second surgery |
| Balamoun (2010) | 25/M | 4-day LUQ pain, obstipation, vomiting | X-ray/contrast: cecal volvulus; reversed positions of colon & duodenum | Reversed midgut rotation | Laparotomy: right hemicolectomy + side-to-side ileocolic anastomosis (anterior to duodenum) | Uneventful recovery |
| Deniffel et al. (2018) | 23‑year‑old woman | Recurrent abdominal pain without obstruction | CT: Reversed midgut rotation with proximal jejunum and transverse colon coursing *behind* the SMA (retro-arterial course) | Reversed midgut rotation variant | None — managed conservatively with IV fluids and analgesia | Full recovery without surgery |
| Sözen & Güzel (2012) | 60‑year‑old man | Acute abdominal pain, bilious vomiting | X-ray/CT: Small bowel loops with malposition; volvulus confirmed intraoperatively | Classic malrotation with midgut volvulus | Emergency laparotomy: Detorsion of volvulus and resection of ~30 cm necrotic small bowel | Uneventful recovery after bowel resection |

References:

1-Deniffel, D., Goerke, S.M., Rummeny, E.J. *et al.* Novel variant of reversed midgut rotation – retro-arterial proximal jejunum and transverse colon: a case report and review of the literature. *J Med Case Reports* 12, 261 (2018). <https://doi.org/10.1186/s13256-018-1802-0>

2- Mizuta N, Kikuchi T, Fukuda Y. Adult Intestinal Malrotation Treated with Laparoscopic Ladd Procedure. *Case Rep Surg*. 2022;2022:6874885. Published 2022 Oct 18. doi:10.1155/2022/6874885

3-Sözen S, Güzel K. Intestinal malrotation in an adult: case report. *Ulus Travma Acil Cerrahi Derg*. 2012;18(3):280-282. doi:10.5505/tjtes.2012.60973

4-Glosser LD, Lombardi CV, Knauss HM, Rivero R, Liu S, Jones TJ. Case report of congenital intestinal malrotation in an adult discovered three months status-post appendectomy. *Int J Surg Case Rep*. 2022;91:106795. doi:10.1016/j.ijscr.2022.106795

5-Aregawi AB, Geremew TT, Legese AT, Bahru TT. A rare case of adult intestinal malrotation: A case report. *Int J Surg Case Rep*. 2025;127:110848. doi:10.1016/j.ijscr.2025.110848

6-Aljamal M, Jaber B, Shakhshir A. Intestinal malrotation in a female adult: Case report and literature review. *Radiol Case Rep*. 2025;20(7):3592-3597. Published 2025 May 8. doi:10.1016/j.radcr.2025.04.025

7-Yin MD, Hao LL, Li G, Li YT, Xu BL, Chen XR. Adult-onset congenital intestinal malrotation: A case report and literature review. *Medicine (Baltimore)*. 2024;103(8):e37249. doi:10.1097/MD.0000000000037249

8-Grassi C, Conti L, Palmieri G, et al. Ladd's band in the adult, an unusual case of occlusion: Case report and review of the literature. *Int J Surg Case Rep*. 2020;71:45-49. doi:10.1016/j.ijscr.2020.04.046

9-Islam S, Mahabir AH, Harkissoon R, Ramnarine M, Harnarayan P. Adult Intestinal Malrotation With Congenital Transverse Meso-Colic Internal Hernia: An Infrequent Cause of Small Bowel Obstruction. *Cureus*. 2024;16(6):e63063. Published 2024 Jun 24. doi:10.7759/cureus.63063

10-Naddouri J, Khouah R, Sekkat H, et al. Small bowel obstruction in adults, Ladd's band is an exceptional cause: a case report. *Pan Afr Med J*. 2024;47:34. Published 2024 Jan 26. doi:10.11604/pamj.2024.47.34.36435

11-Akomea-Agyin E, Agbedinu K, Dally CK, Galley F, Kankam EO, Banini GE. Duodenal stenosis in adult malrotation: When Ladd procedure is not enough: A case report. *Int J Surg Case Rep*. 2024;119:109713. doi:10.1016/j.ijscr.2024.109713

12-Balamoun H, Mohammad R, Hamade A. Reversed rotation of the midgut in adults--a case report. *Ann R Coll Surg Engl*. 2010;92(7):W3-W5. doi:10.1308/147870810X12822015504446

**Note:The references listed in this table are specific to the literature review presented herein, except for two references which are also cited in the main article [**Deniffel et al. (2018), Sözen & Güzel (2012)]
